# Longitudinal trajectories of nutrition-related biomarkers and mortality risk in maintenance hemodialysis patients: a joint modeling analysis

**DOI:** 10.3389/fnut.2026.1769563

**Published:** 2026-06-24

**Authors:** Min-Jia Li, Hong-Yong Liu, Yun-Qiang Zhang, Jian-Hong Zhang, Sheng-Rong Li, Xiu-Lan Yang, Yu Zeng

**Affiliations:** Department of Nephrology, The Third Affiliated Hospital of Sun Yat-Sen University, Yuedong Hospital, Meizhou, Guangdong, China

**Keywords:** hemodialysis, joint modeling, longitudinal analysis, malnutrition, mortality, nutrition-related biomarkers

## Abstract

**Background:**

Malnutrition and nutrition-related abnormalities are common in people with kidney failure, particularly among patients receiving maintenance hemodialysis, and are associated with adverse clinical outcomes. Nutritional status and related biological processes are dynamic; however, the prognostic relevance of their longitudinal changes remains incompletely understood.

**Objective:**

To investigate the associations between longitudinal trajectories of nutrition-related biomarkers and all-cause mortality in patients receiving maintenance hemodialysis using joint modeling.

**Methods:**

This retrospective longitudinal cohort study included 345 maintenance hemodialysis patients, among whom 99 deaths occurred during follow-up. Longitudinal changes in nutrition-related biomarkers were analyzed in relation to all-cause mortality using joint models that integrate repeated biomarker measurements with time-to-event outcomes. Dynamic prediction was evaluated using a fixed 12-month prediction horizon.

**Results:**

In adjusted univariable joint models, several longitudinal biomarkers were associated with all-cause mortality. In the primary multivariable joint model incorporating longitudinal C-reactive protein (CRP) and serum iron, the current value of CRP was positively associated with mortality risk (HR, 1.030; 95% credible interval [CrI], 1.001–1.058), as was the current value of serum iron (HR, 1.073; 95% CrI, 1.015–1.132). Across landmark times from 6 to 36 months, time-dependent AUC values ranged from 0.756 to 0.807 for 12-month dynamic prediction.

**Conclusion:**

The current values of CRP and serum iron were positively associated with all-cause mortality in maintenance hemodialysis patients. Joint modeling of repeated biomarker measurements may help characterize dynamic nutrition-related inflammatory and metabolic risk in kidney failure.

## Introduction

Malnutrition and nutrition-related abnormalities are common clinical challenges in people with kidney failure and are particularly prevalent among patients receiving maintenance hemodialysis (1). As kidney disease progresses, complex disturbances in appetite, metabolism, and nutrient utilization frequently emerge, contributing to deterioration in nutritional status (2). These nutrition-related problems have been consistently associated with adverse clinical outcomes, including increased mortality, higher hospitalization rates, and reduced quality of life in hemodialysis populations (3–5).

Given the growing number of patients living with kidney failure worldwide (6), understanding factors related to nutritional status has become a central focus of clinical nutrition research. In the context of hemodialysis, nutritional vulnerability is often multifactorial and may evolve over time, underscoring the importance of evaluating nutrition-related processes rather than relying solely on static assessments (7).

Nutritional disturbances in kidney failure rarely occur in isolation and are often accompanied by chronic inflammation and metabolic derangements (8). In patients undergoing hemodialysis, inflammation, altered energy metabolism, and dysregulated iron homeostasis frequently coexist and may interact with nutritional status (9). As a result, routinely measured clinical biomarkers reflecting inflammatory and metabolic processes are commonly used to characterize the broader biological milieu associated with nutritional vulnerability (10).

Markers such as C-reactive protein (CRP) and indicators of iron metabolism are widely available in clinical practice and reflect processes closely linked to nutrition-related inflammatory and metabolic stress in kidney failure. While these biomarkers are not direct measures of malnutrition, they provide insight into biological conditions that may influence nutritional status and long-term outcomes. However, how changes in such nutrition-related biomarkers over time relate to prognosis in hemodialysis patients remains incompletely understood.

Despite growing interest in nutrition-related factors in kidney failure, several important gaps remain in the existing literature. Most studies evaluating nutritional or inflammation-related biomarkers in hemodialysis patients have relied on single baseline measurements, which may not adequately capture the evolving nature of nutritional and metabolic disturbances over time (11, 12). Given the dynamic clinical course of patients receiving maintenance hemodialysis, such static assessments may provide an incomplete representation of nutrition-related risk (13).

Furthermore, relatively few studies have examined longitudinal changes in nutrition-related biomarkers in relation to hard clinical outcomes, such as all-cause mortality. Even when repeated measurements are available, they are often analyzed separately from survival outcomes, limiting the ability to assess how temporal biomarker patterns relate to prognosis. As a result, the prognostic relevance of dynamic nutrition-related biological processes in hemodialysis patients remains insufficiently characterized.

## Methodology

### Study design and study population

**Figure 1:**
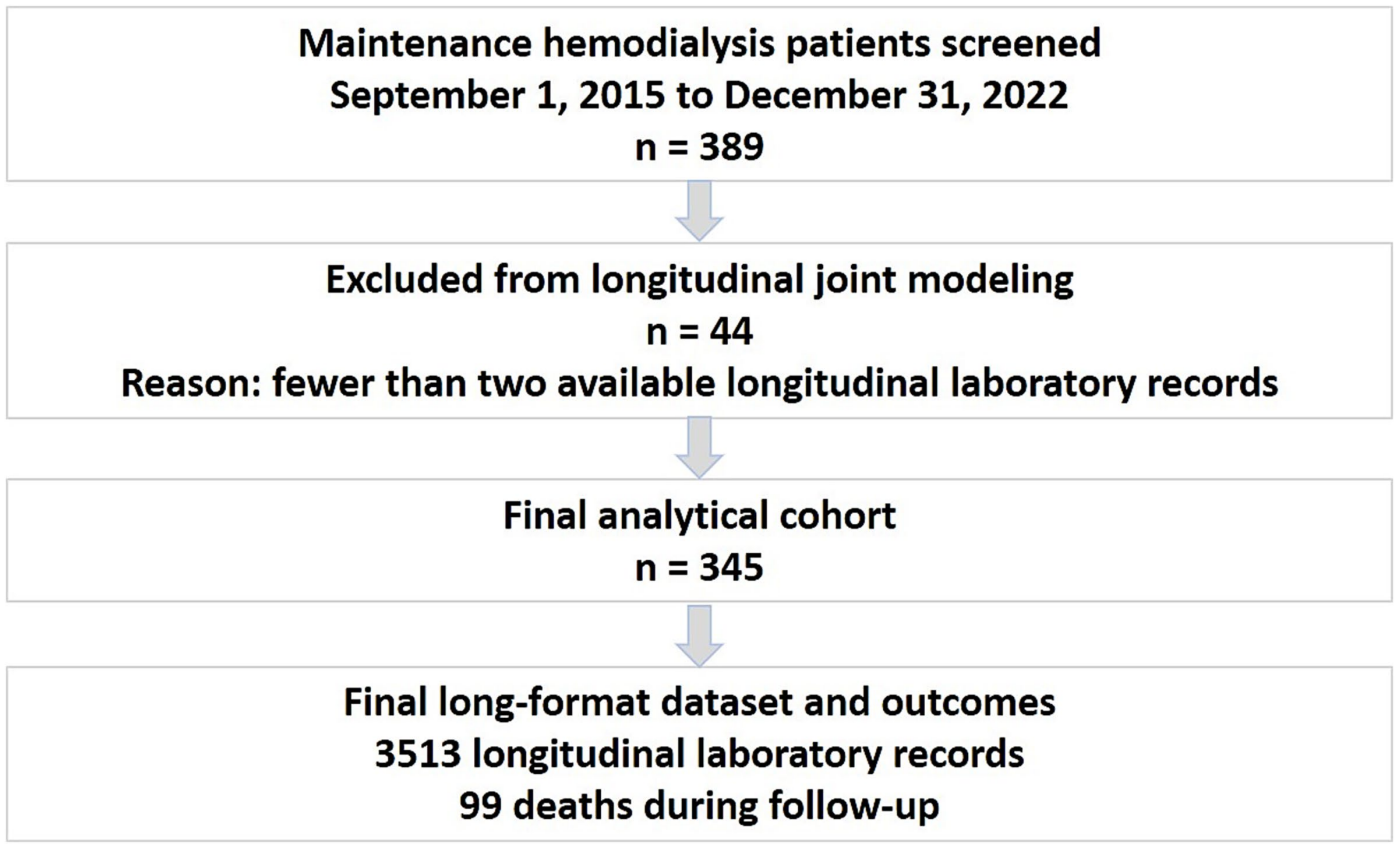
Study flow diagram. A total of 389 patients receiving maintenance hemodialysis during the study period were screened. Forty-four patients were excluded because fewer than two longitudinal laboratory records were available. The final analytical cohort included 345 patients, contributing 3,513 long-format laboratory records, with 99 deaths during follow-up.

This retrospective longitudinal cohort study was conducted at the Blood Purification Center of the Third Affiliated Hospital of Sun Yat-sen University, Yuedong Hospital (Meizhou, Guangdong, China). Patients receiving maintenance hemodialysis between September 1, 2015, and December 31, 2022 were screened for eligibility. The flow of patient selection and construction of the final analytical cohort is summarized in Figure 1.

Eligible participants were adult patients receiving maintenance hemodialysis who had repeated laboratory measurements during follow-up and ascertainable survival outcome information. Among 389 screened patients, 44 were excluded because fewer than two longitudinal laboratory records were available and longitudinal biomarker trajectories could therefore not be evaluated. The final analytical cohort consisted of 345 patients, all of whom were included in baseline description and longitudinal survival analyses.

This study complied with the Declaration of Helsinki and received approval from the Ethics Committee of the Third Affiliated Hospital of Sun Yat-sen University, Yuedong Hospital (approval ID: II2024-024-01). Owing to the retrospective design and the use of anonymized clinical records, the requirement for written informed consent was waived. No additional interventions were performed, and no vulnerable populations were enrolled.

### Data collection and variable definitions

Baseline demographic and clinical characteristics were extracted from the electronic medical record system at study entry, including age, sex, dialysis frequency, baseline dialysis vintage, and comorbid conditions such as diabetes mellitus. Dialysis frequency was recorded as the number of hemodialysis sessions per week and was also summarized categorically as ≥3 sessions per week versus <3 sessions per week for descriptive analyses. Baseline dialysis vintage was defined as dialysis duration at study entry.

Longitudinal laboratory data were obtained during routine clinical follow-up as part of standard care. Biomarkers representing nutritional status, inflammation, hematologic indices, metabolic parameters, and mineral metabolism were included, comprising serum albumin, hemoglobin, C-reactive protein (CRP), serum iron, ferritin, total iron-binding capacity (TIBC), transferrin saturation (TSAT), blood urea nitrogen, creatinine, uric acid, potassium, calcium, phosphorus, parathyroid hormone, carbon dioxide combining power, glucose, total cholesterol, and low-density lipoprotein (LDL) cholesterol. All laboratory measurements were performed in the hospital’s central laboratory using standardized assays and recorded in consistent units.

For longitudinal analyses, repeated biomarker measurements were organized in long format, indexed by patient identifier and follow-up time. Follow-up time was defined as the interval (in months) from baseline to each laboratory measurement. Baseline laboratory values were defined as the first available measurement at study entry.

### Outcome definition

The primary outcome was all-cause mortality during follow-up. Survival time was calculated from baseline to the date of death or censoring, whichever occurred first. Patients who were alive at the end of follow-up were censored at the time of the last available clinical contact or laboratory assessment. Time was measured in months.

### Baseline statistical analysis

Baseline characteristics were summarized using descriptive statistics. Continuous variables were expressed as medians with interquartile ranges (IQRs), and categorical variables were presented as counts and percentages. Group comparisons were performed using the Wilcoxon rank-sum test for continuous variables and the Pearson chi-squared test or Fisher’s exact test for categorical variables, as appropriate.

### Cox proportional hazards regression analysis

Univariable Cox proportional hazards regression analyses were performed to examine associations between baseline variables and all-cause mortality. Each baseline variable was entered into a separate Cox model. Hazard ratios (HRs) with corresponding 95% confidence intervals (CIs) were estimated. For continuous variables, HRs were interpreted per one-unit increase unless otherwise specified. Male sex, diabetes mellitus, and dialysis frequency <3 sessions per week were modeled as binary variables, with female sex, absence of diabetes mellitus, and dialysis frequency ≥3 sessions per week as the reference categories, respectively. Proportional hazards assumptions were assessed using standard diagnostic approaches. Cox regression analyses were conducted to provide a conventional survival analysis framework for baseline variables and to facilitate comparison with joint model results.

### Joint modeling of longitudinal biomarkers and survival

Longitudinal trajectories of repeated laboratory biomarkers were modeled using linear mixed-effects models, incorporating fixed effects to capture average temporal trends and subject-specific random effects to account for within-individual correlation and between-individual heterogeneity. Time was treated as a continuous variable corresponding to follow-up duration in months.

The time-to-event process was modeled using a Cox proportional hazards model, incorporating prespecified baseline covariates. For adjusted joint models, the survival submodel included age, sex, diabetes mellitus, and dialysis frequency. The longitudinal and survival submodels were linked using a current value association structure, whereby the instantaneous hazard of mortality depended on the underlying true value of the longitudinal biomarker at the same time point. The strength of this association was quantified by an association parameter (*α*). This joint modeling framework accounts for measurement error in longitudinal biomarkers and informative dropout due to death (14).

### Strategy for evaluating candidate longitudinal biomarkers

A two-stage strategy was applied to evaluate candidate longitudinal biomarkers. First, separate unadjusted univariable joint models were fitted for each biomarker. Second, adjusted univariable joint models were fitted using a consistent adjustment set in the survival submodel while maintaining identical longitudinal submodel specifications and association structures. The adjusted univariable joint models included age, sex, diabetes mellitus, and dialysis frequency in the survival submodel. No correction for multiple testing was applied at this exploratory stage; results were used solely to inform the construction of candidate multivariable joint models. Based on the adjusted univariable joint model results and the clinical relevance of inflammation and iron metabolism, C-reactive protein and serum iron were selected for the primary multivariable joint model.

### Sensitivity analysis and dynamic prediction performance

As a sensitivity analysis, an additional multivariable joint model was fitted by further adjusting the survival submodel for baseline dialysis vintage, defined as dialysis duration at study entry. Dynamic prediction performance was evaluated using the primary adjusted multivariable joint model incorporating longitudinal C-reactive protein and serum iron. Time-dependent AUC and Brier score were calculated at landmark times of 6, 9, 12, 15, 18, 21, 24, 27, 30, 33, and 36 months, using a fixed 12-month prediction horizon. At each landmark time, survival probabilities over the subsequent 12 months were estimated conditional on the longitudinal biomarker history available up to that time.

### Software and statistical environment

All statistical analyses were performed using R software (version 4.3.2). Baseline descriptive analyses and Cox regression models were fitted using the survival package. Linear mixed-effects models were fitted using nlme, and joint models were fitted using JMbayes2. Data management and visualization were performed using data.table and ggplot2.

## Results

### Baseline characteristics of the study population

A total of 345 maintenance hemodialysis patients were included in the final analytical cohort, of whom 246 survived and 99 died during follow-up (Table 1).

**Table 1:** Baseline characteristics of the study population stratified by survival status.

Characteristic	Total (*n* = 345)	Survivors (*n* = 246)	Deceased (*n* = 99)	*p*-value
Age, years	64 [56, 73]	61 [54, 69]	71 [63, 77]	<0.001
Sex, male	220 (63.8%)	153 (62.2%)	67 (67.7%)	0.338
Diabetes mellitus	143 (41.4%)	88 (35.8%)	55 (55.6%)	<0.001
Baseline dialysis vintage, years	0.4 [0.2, 0.8]	0.3 [0.2, 0.6]	0.6 [0.4, 1.0]	<0.001
Dialysis frequency, sessions/week	2.0 [2.0, 3.0]	2.0 [2.0, 2.5]	2.0 [2.0, 3.0]	0.874
Dialysis frequency <3 sessions/week	256 (74.2%)	186 (75.6%)	70 (70.7%)	0.346
Albumin, g/L	37.4 [35.1, 39.8]	37.8 [35.3, 40.0]	36.6 [33.8, 39.4]	0.022
Hemoglobin, g/L	106 [92, 119]	108 [95, 120]	100 [84, 113]	<0.001
C-reactive protein, mg/L	4.0 [2.0, 9.0]	4.0 [2.0, 8.2]	5.0 [3.0, 12.0]	0.016
Serum iron, μmol/L	11.3 [8.6, 14.4]	10.8 [8.5, 13.8]	12.5 [9.5, 16.4]	0.001
Ferritin, ng/mL	158.4 [70.6, 369.3]	170.8 [74.1, 381.6]	134.3 [67.1, 297.0]	0.225
Total iron-binding capacity, μmol/L	40.6 [35.5, 47.5]	40.5 [36.0, 48.4]	41.0 [34.9, 46.1]	0.484
Transferrin saturation, %	26.5 [19.8, 36.1]	27.3 [21.1, 36.0]	23.6 [18.4, 37.9]	0.174
Blood urea nitrogen, mmol/L	26.9 [22.1, 32.3]	27.5 [22.2, 31.5]	26.8 [21.2, 32.9]	0.403
Creatinine, μmol/L	954.7 [776.1, 1248.1]	960.4 [800.9, 1254.6]	942.0 [741.7, 1247.9]	0.223
Uric acid, μmol/L	488 [428, 558]	485 [429, 559]	494 [422, 539]	0.705
Potassium, mmol/L	5.08 [4.51, 5.60]	5.06 [4.52, 5.61]	5.09 [4.50, 5.52]	0.692
Calcium, mmol/L	2.14 [1.99, 2.31]	2.14 [1.97, 2.31]	2.12 [2.01, 2.31]	0.745
Phosphorus, mmol/L	2.01 [1.68, 2.48]	2.01 [1.66, 2.44]	2.00 [1.72, 2.53]	0.753
Parathyroid hormone, pg/mL	331.0 [194.3, 498.0]	342.8 [200.0, 519.8]	300.0 [180.0, 444.3]	0.165
Carbon dioxide combining power, mmol/L	17.8 [15.4, 20.3]	18.1 [15.6, 20.5]	16.9 [15.0, 19.4]	0.008
Glucose, mmol/L	7.70 [6.20, 10.57]	7.62 [6.15, 10.04]	8.08 [6.23, 11.31]	0.423
Total cholesterol, mmol/L	3.94 [3.38, 4.83]	4.03 [3.43, 4.91]	3.82 [3.20, 4.57]	0.07
Low-density lipoprotein cholesterol, mmol/L	1.94 [1.52, 2.53]	2.01 [1.56, 2.56]	1.84 [1.44, 2.30]	0.074

Data are presented as median [IQR] for continuous variables and *n* (%) for categorical variables. *p*-values compare survivors and deceased patients using the Wilcoxon rank-sum test for continuous variables and Pearson’s chi-squared test or Fisher’s exact test for categorical variables, as appropriate. IQR, interquartile range.

Patients who died were older than survivors (median age: 71 vs. 61 years) and had a higher prevalence of diabetes mellitus (55.6% vs. 35.8%). Deceased patients also had a longer baseline dialysis vintage than survivors (median: 0.6 vs. 0.3 years). At baseline, deceased patients had lower albumin and hemoglobin levels and higher C-reactive protein (CRP) and serum iron levels compared with survivors. Sex distribution and dialysis frequency did not differ significantly between survivors and deceased patients.

### Results of univariable cox regression analysis

Results of univariable Cox proportional hazards regression analyses for baseline variables are presented in Table 2. Older age and the presence of diabetes mellitus were associated with an increased risk of all-cause mortality. Among baseline laboratory variables, higher levels of C-reactive protein and serum iron were associated with increased mortality risk, whereas higher albumin and hemoglobin levels were associated with lower mortality risk.

**Table 2:** Univariable Cox proportional hazards analysis of baseline variables for all-cause mortality.

Variable	HR (95% CI)	*P*-value
Age, per year	1.067 (1.047 to 1.088)	<0.001
Male sex	1.256 (0.824 to 1.914)	0.29
Diabetes mellitus	2.234 (1.496 to 3.336)	<0.001
Baseline dialysis vintage, per year	0.958 (0.874 to 1.050)	0.358
Dialysis frequency, per 1 session/week increase	0.675 (0.458 to 0.995)	0.047
Dialysis frequency <3 sessions/week	1.083 (0.702 to 1.671)	0.719
Albumin, per g/L	0.915 (0.873 to 0.960)	<0.001
Hemoglobin, per g/L	0.986 (0.977 to 0.996)	0.005
C-reactive protein, per mg/L	1.020 (1.011 to 1.030)	<0.001
Serum iron, per μmol/L	1.046 (1.017 to 1.076)	0.002
Ferritin, per ng/mL	1.000 (0.999 to 1.000)	0.543
Total iron-binding capacity, per μmol/L	0.971 (0.950 to 0.992)	0.006
Transferrin saturation, per %	1.008 (0.996 to 1.020)	0.192
Blood urea nitrogen, per mmol/L	0.986 (0.960 to 1.013)	0.317
Creatinine, per μmol/L	0.999 (0.999 to 1.000)	0.003
Uric acid, per μmol/L	0.999 (0.997 to 1.001)	0.175
Potassium, per mmol/L	0.911 (0.709 to 1.169)	0.462
Calcium, per mmol/L	0.633 (0.292 to 1.373)	0.247
Phosphorus, per mmol/L	0.837 (0.624 to 1.122)	0.234
Parathyroid hormone, per pg/mL	0.999 (0.998 to 1.000)	0.017
Carbon dioxide combining power, per mmol/L	0.979 (0.922 to 1.039)	0.477
Glucose, per mmol/L	1.023 (0.982 to 1.065)	0.284
Total cholesterol, per mmol/L	0.862 (0.734 to 1.013)	0.071
Low-density lipoprotein cholesterol, per mmol/L	0.750 (0.575 to 0.978)	0.034

Univariable Cox proportional hazards models were fitted separately for each baseline variable. Hazard ratios are presented with 95% confidence intervals. For continuous variables, hazard ratios are presented per one-unit increase unless otherwise specified. Male sex, diabetes mellitus, and dialysis frequency <3 sessions/week were modeled as binary variables, with female sex, absence of diabetes mellitus, and dialysis frequency ≥3 sessions/week as the reference categories. CI, confidence interval; HR, hazard ratio.

Baseline dialysis vintage was not associated with all-cause mortality in univariable Cox analysis. Dialysis frequency showed a weak inverse association with mortality when modeled as a continuous variable, whereas the binary indicator of dialysis frequency <3 sessions per week was not associated with mortality. Additional univariable associations for other biochemical variables are shown in Table 2 and should be interpreted as exploratory.

### Evaluation of longitudinal nutritional biomarkers using joint models

Results of adjusted univariable joint models evaluating associations between longitudinal biomarkers and all-cause mortality are summarized in Table 3. Several longitudinal biomarkers were associated with all-cause mortality. Albumin and hemoglobin showed inverse longitudinal–survival associations, whereas C-reactive protein (CRP) and serum iron showed positive associations. Specifically, longitudinal CRP was positively associated with mortality risk (association parameter *α* = 0.0399, 95% credible interval (CrI) 0.0122 to 0.0651), and longitudinal serum iron was also positively associated with mortality (*α* = 0.0901, 95% CrI 0.0380 to 0.1392).

**Table 3:** Adjusted univariable joint models evaluating longitudinal biomarkers associated with all-cause mortality.

Biomarker	*N* observations	Association parameter α (95% CrI)	*P*-value
Albumin, g/L	3,513	−0.0989 (−0.1948 to −0.0008)	0.049
Hemoglobin, g/L	3,513	−0.0223 (−0.0381 to −0.0061)	0.012
C-reactive protein, mg/L	3,512	0.0399 (0.0122 to 0.0651)	0.009
Serum iron, μmol/L	3,512	0.0901 (0.0380 to 0.1392)	<0.001
Ferritin, ng/mL	3,513	−0.0007 (−0.0017 to 0.0002)	0.103
Total iron-binding capacity, μmol/L	3,512	0.0209 (−0.0172 to 0.0570)	0.251
Transferrin saturation, %	3,498	−0.0078 (−0.0480 to 0.0296)	0.686
Blood urea nitrogen, mmol/L	3,513	−0.0050 (−0.0456 to 0.0343)	0.813
Creatinine, μmol/L	3,513	−0.0011 (−0.0020 to −0.0003)	0.018
Uric acid, μmol/L	3,513	−0.0015 (−0.0050 to 0.0019)	0.404
Potassium, mmol/L	3,513	−0.0536 (−0.4250 to 0.3125)	0.777
Calcium, mmol/L	3,513	−0.5170 (−1.8509 to 0.8296)	0.447
Phosphorus, mmol/L	3,513	0.0849 (−0.4140 to 0.6042)	0.747
Parathyroid hormone, pg/mL	3,513	−0.0003 (−0.0009 to 0.0003)	0.305
Carbon dioxide combining power, mmol/L	3,513	0.0009 (−0.0936 to 0.0962)	0.971
Glucose, mmol/L	3,512	0.0105 (−0.0612 to 0.0784)	0.752
Total cholesterol, mmol/L	3,513	−0.4242 (−0.7303 to −0.1289)	0.007
Low-density lipoprotein cholesterol, mmol/L	3,513	−0.4810 (−0.8996 to −0.0765)	0.019

Each joint model included one longitudinal biomarker and adjusted for age, sex, diabetes mellitus, and dialysis frequency in the survival submodel. Association parameters are presented on the log-hazard scale as posterior means with 95% credible intervals. All models included 345 patients; the number of longitudinal observations available for each biomarker is shown. Statistical significance was not adjusted for multiple testing because this stage was exploratory and used to inform candidate multivariable joint models. CrI, credible interval.

Creatinine, total cholesterol, and low-density lipoprotein (LDL) cholesterol also showed inverse associations with mortality in adjusted univariable joint models. Because this stage was exploratory and not adjusted for multiple testing, these findings were used to inform candidate multivariable joint modeling rather than to establish independent prognostic effects.

Based on the adjusted univariable joint model results and the clinical relevance of inflammation and iron metabolism, CRP and serum iron were selected for the primary multivariable joint model.

### Multivariable joint model of longitudinal biomarkers and mortality

**Figure 2:**
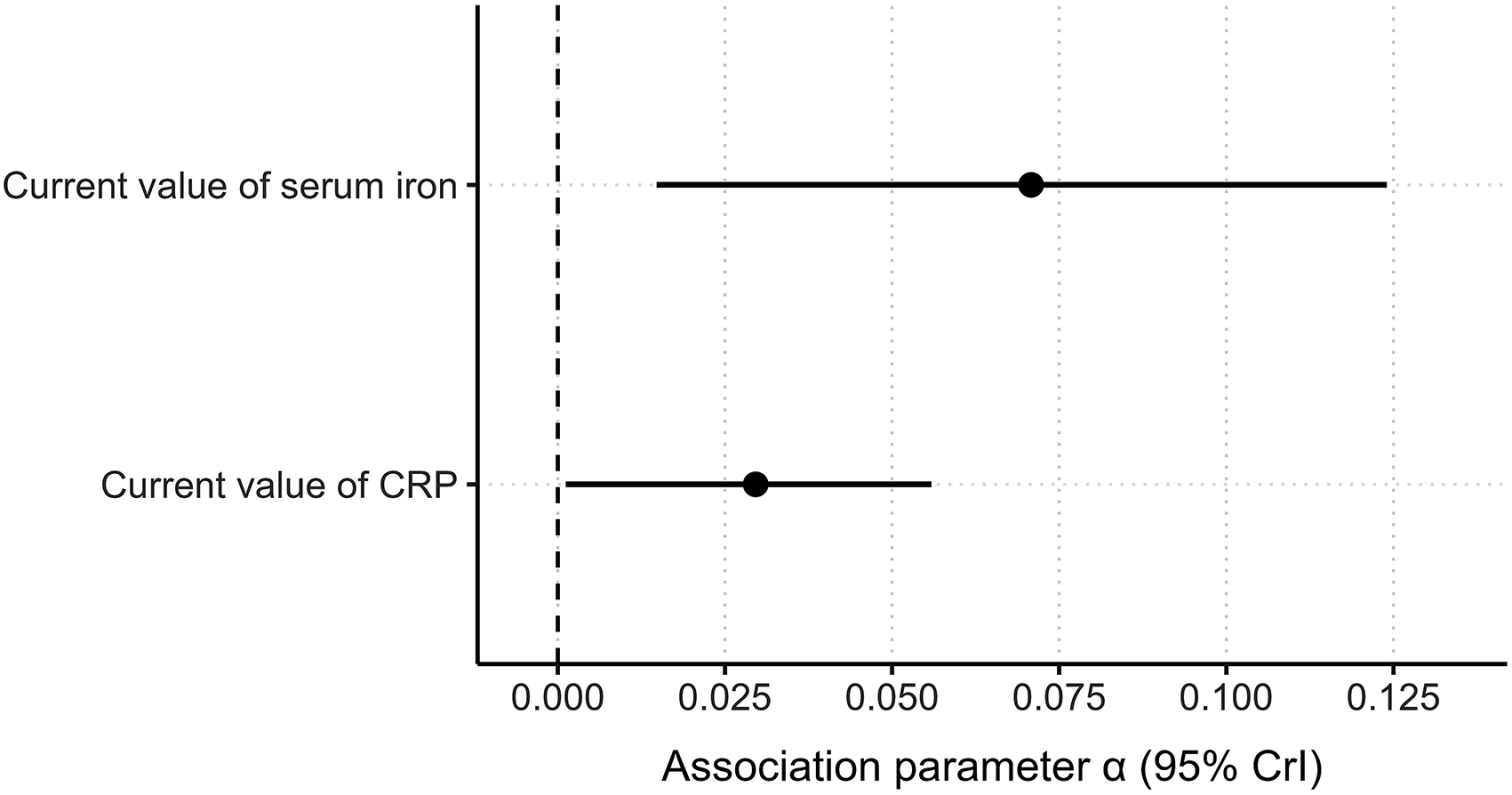
Association parameters from the adjusted joint model. Points indicate posterior mean estimates of the association parameters, and horizontal lines indicate 95% credible intervals. The adjusted joint model incorporated longitudinal C-reactive protein and serum iron and linked their current values with all-cause mortality risk. The vertical dashed line indicates the null value of zero. CRP, C-reactive protein; CrI, credible interval.

Results of the adjusted multivariable joint model incorporating longitudinal C-reactive protein (CRP) and serum iron are presented in Table 4 and Figure 2. In the survival submodel, older age and diabetes mellitus were associated with higher all-cause mortality risk, whereas sex and dialysis frequency were not clearly associated with mortality.

**Table 4:** Adjusted multivariable joint model incorporating longitudinal C-reactive protein and serum iron.

Section	Parameter	Estimate (95% CrI)	HR (95% CrI)	*P*-value
Survival submodel	Age, per year	0.0556 (0.0311 to 0.0794)	1.057 (1.032 to 1.083)	<0.001
Survival submodel	Male sex	0.2296 (−0.2318 to 0.6757)	1.258 (0.793 to 1.966)	0.349
Survival submodel	Diabetes mellitus	0.7708 (0.3509 to 1.2177)	2.162 (1.420 to 3.379)	<0.001
Survival submodel	Dialysis frequency, per 1 session/week increase	−0.2276 (−0.5980 to 0.1561)	0.796 (0.550 to 1.169)	0.225
Longitudinal-survival association	Current value of CRP	0.0296 (0.0012 to 0.0559)	1.030 (1.001 to 1.058)	0.041
Longitudinal-survival association	Current value of serum iron	0.0708 (0.0148 to 0.1240)	1.073 (1.015 to 1.132)	0.011

Values are posterior means with 95% credible intervals unless otherwise indicated. Estimates are presented on the log-hazard scale. Hazard ratios were calculated by exponentiating the corresponding log-hazard coefficients. The survival submodel was adjusted for age, sex, diabetes mellitus, and dialysis frequency. For continuous variables, hazard ratios are presented per one-unit increase. Association parameters quantify the association between the current value of each longitudinal biomarker and the hazard of all-cause mortality. CRP, C-reactive protein; CrI, credible interval; HR, hazard ratio.

In the longitudinal–survival association component, the current value of CRP was positively associated with mortality risk (association parameter *α* = 0.0296, 95% CrI 0.0012 to 0.0559; HR, 1.030; 95% CrI 1.001 to 1.058). The current value of serum iron was also positively associated with mortality risk (*α* = 0.0708, 95% CrI 0.0148 to 0.1240; HR, 1.073; 95% CrI 1.015 to 1.132). Figure 2 illustrates the estimated association parameters and corresponding credible intervals from the adjusted joint model.

### Longitudinal trajectories of key biomarkers

**Figure 3:**
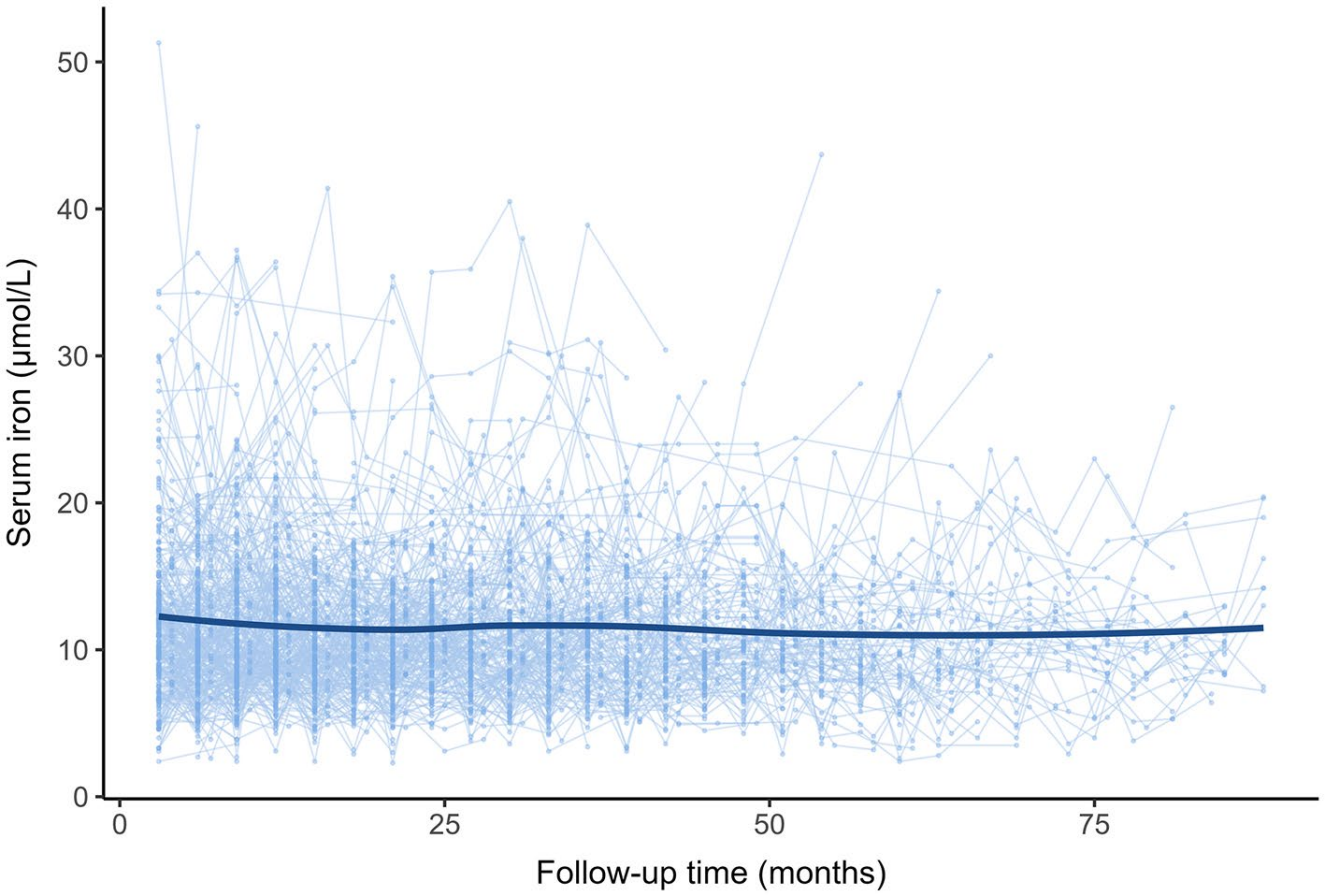
Longitudinal trajectory of serum iron. Thin lines represent individual observed trajectories, and the thick line represents the smoothed overall trend.

**Figure 4:**
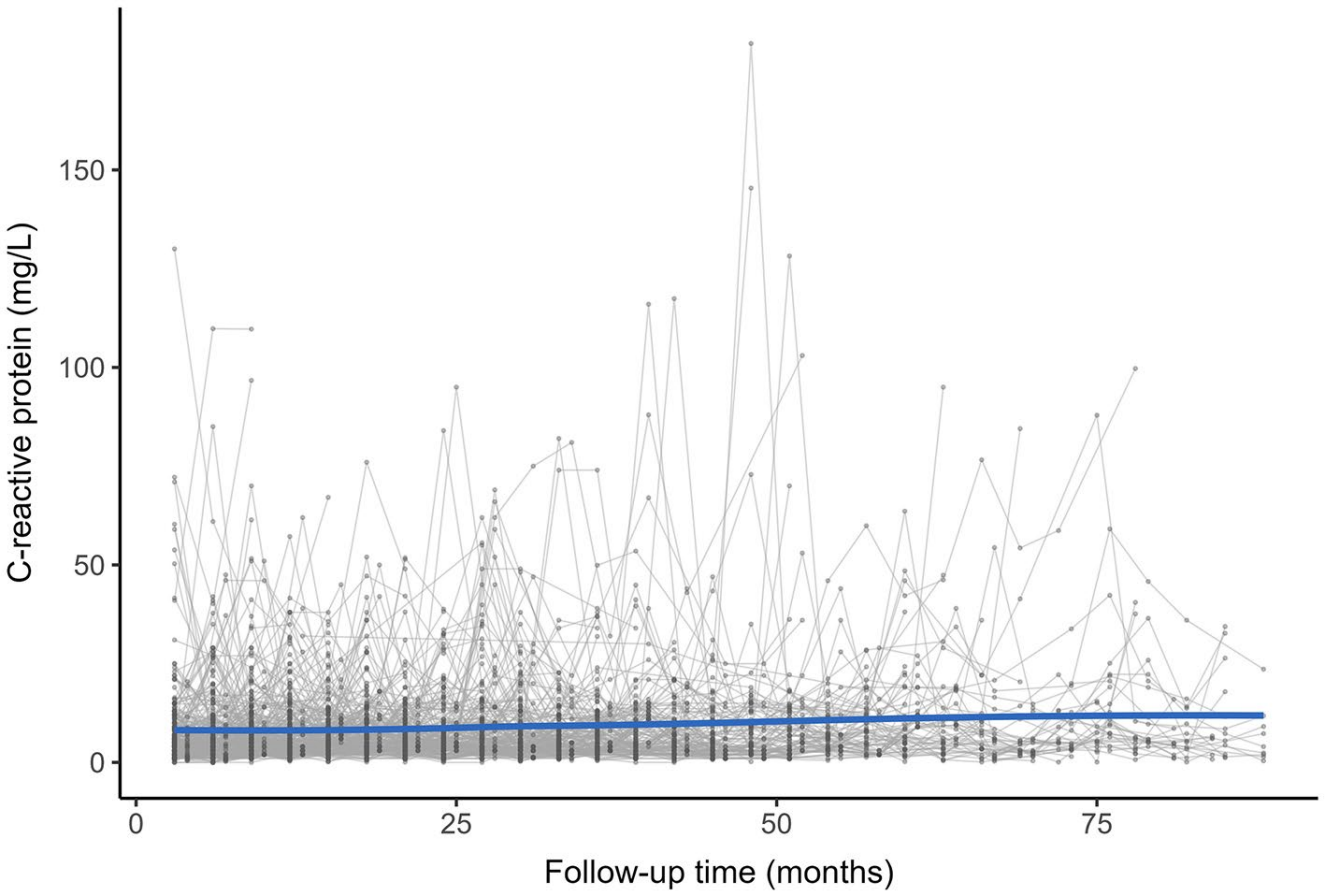
Longitudinal trajectory of C-reactive protein. Thin lines represent individual observed trajectories, and the thick line represents the smoothed overall trend. CRP, C-reactive protein.

Longitudinal trajectories of serum iron and C-reactive protein (CRP) over follow-up are illustrated in Figures 3, 4, respectively. Individual-level trajectories demonstrated substantial heterogeneity across patients, with marked variability in biomarker levels over time. Superimposed smoothed curves depict the overall temporal patterns of CRP and serum iron during follow-up.

### Dynamic prediction performance of the joint model

**Figure 5:**
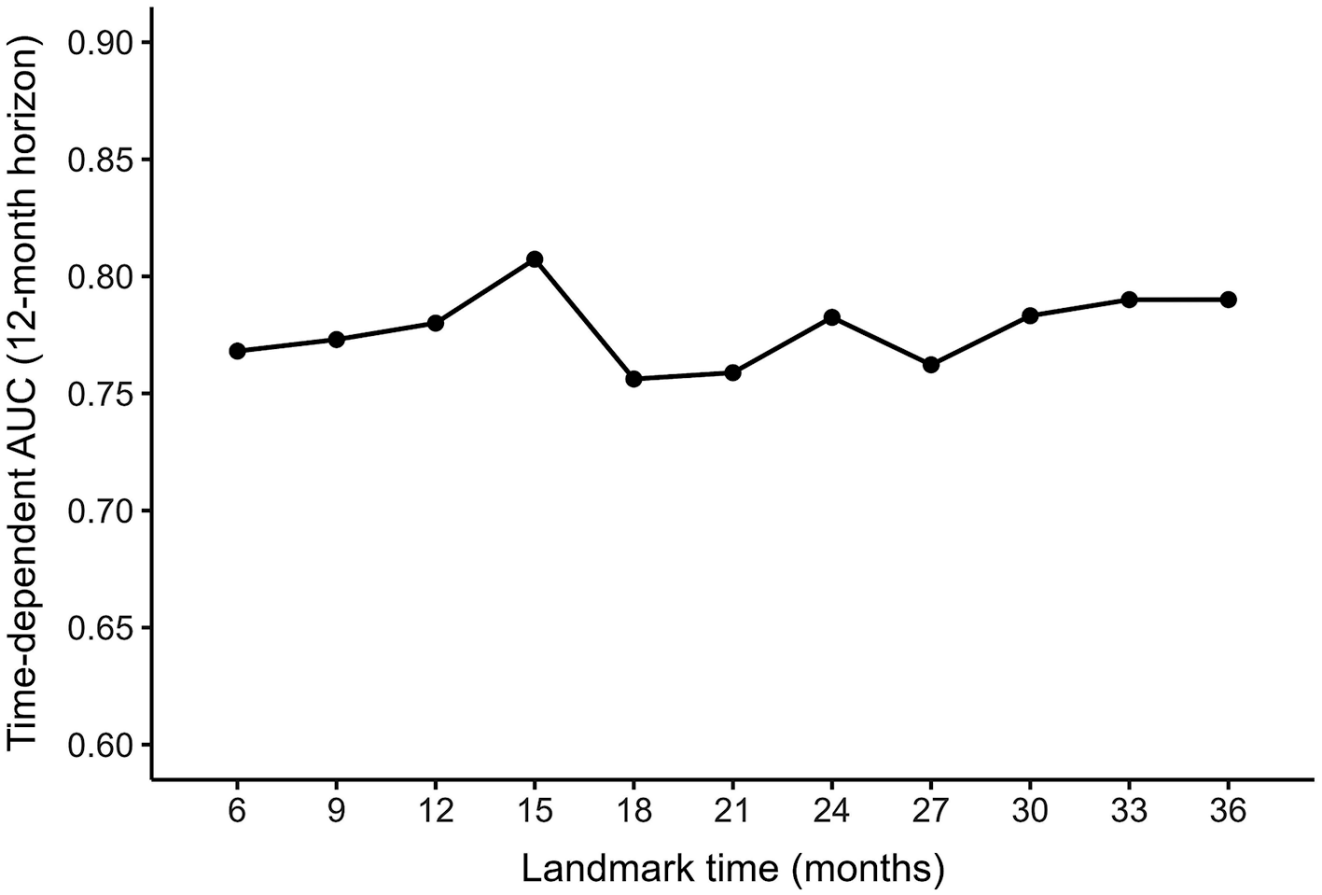
Time-dependent AUC for 12-month dynamic prediction. Time-dependent AUC values were calculated from the primary adjusted joint model incorporating longitudinal C-reactive protein and serum iron, using a fixed 12-month prediction horizon across landmark times from 6 to 36 months. AUC, area under the curve.

**Figure 6:**
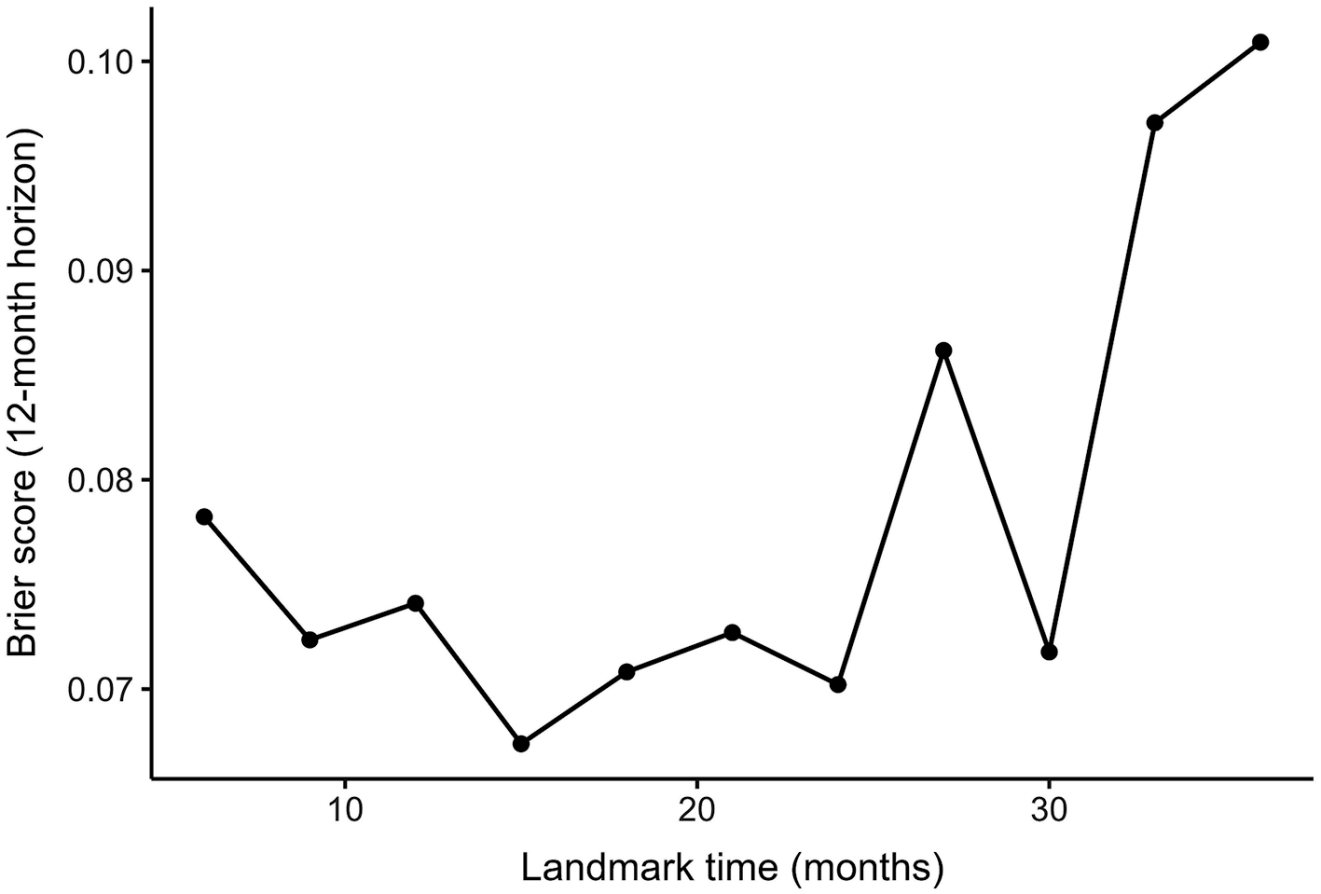
Brier score for 12-month dynamic prediction. Brier scores were calculated from the primary adjusted joint model incorporating longitudinal C-reactive protein and serum iron, using a fixed 12-month prediction horizon across landmark times from 6 to 36 months. Lower Brier scores indicate lower prediction error.

The dynamic predictive performance of the primary adjusted joint model incorporating longitudinal CRP and serum iron was evaluated using a landmarking approach with a fixed 12-month prediction horizon. Time-dependent discrimination and prediction error metrics are shown in Figures 5, 6, with detailed values provided in Supplementary Table S3.

Using the fixed 12-month prediction horizon, the time-dependent AUC ranged from 0.756 to 0.807 across landmark times from 6 to 36 months, indicating moderate dynamic discrimination. The corresponding Brier scores ranged from 0.067 to 0.101, with higher prediction error observed at later landmark times.

### Supplementary analyses

Additional analyses are provided in the Supplementary material to support the main findings. Unadjusted univariable joint models evaluating longitudinal biomarkers are presented in Supplementary Table S1. In the sensitivity joint model, additional adjustment for baseline dialysis vintage did not materially change the associations of current CRP and serum iron with all-cause mortality. Baseline dialysis vintage itself was not clearly associated with mortality, and these sensitivity results are presented in Supplementary Table S2. Detailed time-dependent AUC and Brier score values for the dynamic prediction analysis are provided in Supplementary Table S3.

## Discussion

In this longitudinal cohort of maintenance hemodialysis patients, we observed that trajectories of selected nutrition-related biomarkers were associated with all-cause mortality. Using joint models that simultaneously accounted for repeated measurements and time-to-event outcomes, the current values of C-reactive protein (CRP) and serum iron showed positive associations with mortality risk in the primary multivariable joint model. These findings support interpreting iron-related biomarkers within a broader nutritional, inflammatory, and metabolic context when assessing long-term outcomes in maintenance hemodialysis.

Importantly, this study was not intended to establish CRP or serum iron as diagnostic markers of malnutrition. Rather, these biomarkers were examined as indicators of nutrition-related inflammatory and metabolic stress, which are common in kidney failure and closely intertwined with adverse outcomes. Persistent disturbances in inflammation and iron metabolism may reflect underlying catabolic states and impaired nutritional homeostasis, thereby marking a higher-risk biological state in patients receiving maintenance hemodialysis.

Chronic inflammation is a hallmark of kidney failure and represents a key link between nutritional disturbances and adverse outcomes (15). In patients receiving maintenance hemodialysis, persistent inflammatory activity is associated with increased protein catabolism, impaired appetite, altered energy balance, and resistance to anabolic signaling, thereby predisposing patients to progressive deterioration in nutritional status (16). Within this framework, CRP reflects the burden of nutrition-related inflammatory stress rather than serving as a direct marker of malnutrition (17).

The positive association between the current value of CRP and mortality risk in the joint model underscores the prognostic relevance of sustained inflammatory stress. Persistently elevated inflammation may indicate an ongoing catabolic state that disrupts nutritional homeostasis over time, thereby increasing vulnerability to adverse outcomes in hemodialysis patients (18).

Iron metabolism in hemodialysis patients is closely intertwined with nutritional and inflammatory processes rather than representing an isolated hematologic pathway (19). Beyond its role in erythropoiesis, iron homeostasis is influenced by systemic inflammation, oxidative stress, and metabolic regulation, all of which are commonly disturbed in kidney failure. In this context, serum iron may reflect broader nutrition-related metabolic alterations that accompany chronic disease and catabolic stress (20). However, serum iron should not be interpreted as a standalone indicator of iron overload or nutritional reserve, because circulating iron levels may be influenced by inflammation-related iron redistribution and by treatment-related factors in clinical practice.

The positive association between the current value of serum iron and mortality risk in the joint model suggests that disturbances in circulating iron status may carry prognostic information. This association may reflect a mixture of biological processes, including altered iron mobilization, inflammatory dysregulation, oxidative stress, and treatment-related variation, rather than a single causal pathway. Altered iron dynamics may signal underlying metabolic imbalance and inflammation-related dysregulation that coexist with compromised nutritional homeostasis (21). These findings highlight the importance of viewing iron-related biomarkers as part of an integrated nutritional, inflammatory, and metabolic milieu when assessing long-term outcomes in patients receiving maintenance hemodialysis.

Nutritional status in patients with kidney failure is inherently dynamic, reflecting the cumulative and fluctuating effects of inflammation, metabolic derangements, and disease burden over time (22). Single time-point measurements may therefore provide an incomplete representation of nutrition-related risk, particularly in the context of maintenance hemodialysis where clinical and biochemical states evolve continuously. By integrating repeated biomarker measurements with survival outcomes, a longitudinal approach allows nutrition-related biological processes to be evaluated as trajectories rather than isolated values (23).

In the present study, the use of joint modeling highlights the relevance of temporal patterns in nutrition-related biomarkers for prognosis. The trajectory plots of CRP and serum iron further illustrated substantial within-individual variability and between-individual heterogeneity, supporting the need to consider longitudinal biomarker histories rather than isolated measurements. These observations support the use of longitudinal monitoring to capture evolving vulnerability in hemodialysis patients.

The present findings suggest that longitudinal monitoring of nutrition-related biomarkers may offer complementary insight into the evolving risk profile of patients receiving maintenance hemodialysis. Rather than serving as diagnostic tools for malnutrition, trajectories of inflammatory and metabolic markers may help contextualize nutritional vulnerability within the broader disease process. Such information may help identify patients who experience persistent or worsening nutrition-related biological stress over time.

Importantly, these implications should be interpreted as supportive rather than prescriptive. The results do not imply specific nutritional or therapeutic interventions, but instead emphasize the potential value of integrating longitudinal biomarker information into existing nutritional evaluation frameworks to enhance understanding of nutrition-related risk in kidney failure.

Several strengths of this study merit consideration. First, the longitudinal design with repeated biomarker measurements allowed nutrition-related biological processes to be evaluated dynamically rather than relying on single baseline values. Second, the use of joint modeling enabled simultaneous analysis of longitudinal biomarkers and survival outcomes, accounting for measurement error and informative dropout. Third, the study focused on routinely available clinical biomarkers, enhancing the potential relevance of the findings to real-world nutritional assessment in hemodialysis populations. In addition, the analysis incorporated dynamic prediction assessment and a sensitivity joint model adjusted for baseline dialysis vintage, providing additional support for the robustness and transparency of the findings.

This study also has limitations. Its observational nature precludes causal inference, and the single-center design may limit generalizability. The relatively high proportion of patients receiving fewer than three hemodialysis sessions per week may reflect center-specific clinical practice, residual kidney function, socioeconomic or geographic factors, or patient-level adherence, and may therefore limit the representativeness of the cohort. Although dialysis frequency was included as an adjustment covariate, residual confounding related to dialysis adequacy cannot be excluded. Direct measures of nutritional intake, body composition, or formal nutritional assessment tools were not available, and therefore the findings should be interpreted within a nutrition-related biological framework rather than as direct indicators of malnutrition. Reliable repeated data required for longitudinal calculation of composite nutritional indices such as the Geriatric Nutritional Risk Index were not consistently available, and this may have limited a more comprehensive assessment of nutritional trajectories. In addition, detailed longitudinal medication exposure, including intravenous iron therapy, erythropoiesis-stimulating agent dose, and chronic kidney disease–mineral and bone disorder treatment, was not systematically captured. This limitation is particularly relevant to the interpretation of serum iron, because circulating iron levels may be influenced by treatment exposure as well as inflammation-related iron redistribution. Nonetheless, these limitations should be considered alongside the longitudinal approach, the use of joint modeling, and the sensitivity analysis adjusted for baseline dialysis vintage, which together provide a rationale for further studies exploring dynamic nutritional risk assessment in kidney failure.

## Conclusion

In this retrospective longitudinal cohort of maintenance hemodialysis patients, the current values of C-reactive protein and serum iron were positively associated with all-cause mortality in the primary joint model. Joint modeling of repeated biomarker measurements and survival outcomes may help characterize dynamic nutrition-related inflammatory and metabolic risk, highlighting the potential value of longitudinal evaluation of nutrition-related biological processes in kidney failure.

## Data Availability

The data are not publicly available due to patient privacy and institutional restrictions. De-identified data may be available from the corresponding author upon reasonable request and with ethics approval.
