# Supplementary material for: Longitudinal trajectories of nutrition-related biomarkers and mortality risk in maintenance hemodialysis patients: a joint modeling analysis
Source: Front Nutr. 2026 Jun 24;13:1769563. doi: 10.3389/fnut.2026.1769563 (PMC13341511; doi:10.3389/fnut.2026.1769563)
Supplement: Supplementary file 1 [file Data_Sheet_1.PDF]

Supplementary Table S1. Unadjusted univariable joint models evaluating longitudinal biomarkers associated with all-cause mortality

Biomarker	N observations	Association parameter α (95% CrI)	P value
Albumin, g/L	3513	-0.1991 (-0.2960 to -0.0949)	<0.001
Hemoglobin, g/L	3513	-0.0221 (-0.0408 to -0.0017)	0.039
C-reactive protein, mg/L	3512	0.0411 (0.0111 to 0.0684)	0.009
Serum iron, µmol/L	3512	0.0941 (0.0292 to 0.1554)	0.003
Ferritin, ng/mL	3513	-0.0009 (-0.0021 to 0.0003)	0.130
Total iron-binding capacity, µmol/L	3512	0.0012 (-0.0446 to 0.0445)	0.951
Transferrin saturation, %	3498	-0.0118 (-0.0633 to 0.0361)	0.677
Blood urea nitrogen, mmol/L	3513	-0.0385 (-0.0888 to 0.0141)	0.137
Creatinine, µmol/L	3513	-0.0018 (-0.0029 to -0.0007)	0.005
Uric acid, µmol/L	3513	-0.0035 (-0.0081 to 0.0012)	0.139
Potassium, mmol/L	3513	-0.2691 (-0.7637 to 0.2153)	0.287
Calcium, mmol/L	3513	-0.7020 (-2.3239 to 0.9490)	0.416
Phosphorus, mmol/L	3513	-0.4775 (-1.1040 to 0.1564)	0.152
Parathyroid hormone, pg/mL	3513	-0.0008 (-0.0016 to -0.0001)	0.023
Carbon dioxide combining power, mmol/L	3513	0.0584 (-0.0557 to 0.1766)	0.341
Glucose, mmol/L	3512	0.0735 (0.0039 to 0.1452)	0.044
Total cholesterol, mmol/L	3513	-0.5398 (-0.8902 to -0.1713)	<0.001
Low-density lipoprotein cholesterol, mmol/L	3513	-0.6516 (-1.1796 to -0.1123)	0.022

Association parameters (α) represent the strength of association between the current value of each longitudinal biomarker and mortality risk in joint models. Negative values indicate a protective association, whereas positive values indicate increased risk.
