# Supplementary material for: Longitudinal trajectories of nutrition-related biomarkers and mortality risk in maintenance hemodialysis patients: a joint modeling analysis
Source: Front Nutr. 2026 Jun 24;13:1769563. doi: 10.3389/fnut.2026.1769563 (PMC13341511; doi:10.3389/fnut.2026.1769563)
Supplement: Supplementary file 2 [file Data_Sheet_2.PDF]

**Supplementary Table S2. Sensitivity joint model additionally adjusted for baseline dialysis vintage**

| Parameter | Estimate (95% CrI) | HR (95% CrI) | P value |
|---|---|---|---|
| Age, per year | 0.0570 (0.0319 to 0.0819) | 1.059 (1.032 to 1.085) | <0.001 |
| Male sex | 0.2176 (-0.2726 to 0.6939) | 1.243 (0.761 to 2.002) | 0.365 |
| Diabetes mellitus | 0.7838 (0.3631 to 1.2115) | 2.190 (1.438 to 3.359) | <0.001 |
| Dialysis frequency, per 1 session/week increase | -0.2283 (-0.5917 to 0.1418) | 0.796 (0.553 to 1.152) | 0.228 |
| Baseline dialysis vintage, per year | 0.0069 (-0.1088 to 0.1007) | 1.007 (0.897 to 1.106) | 0.826 |
| Current value of CRP | 0.0300 (0.0005 to 0.0567) | 1.030 (1.001 to 1.058) | 0.047 |
| Current value of serum iron | 0.0698 (0.0150 to 0.1220) | 1.072 (1.015 to 1.130) | 0.016 |

Values are posterior means with 95% credible intervals unless otherwise indicated. Estimates are presented on the log-hazard scale. Hazard ratios were calculated by exponentiating the corresponding log-hazard coefficients. The sensitivity joint model incorporated longitudinal C-reactive protein and serum iron and additionally adjusted for baseline dialysis vintage in the survival submodel, along with age, sex, diabetes mellitus, and dialysis frequency. Baseline dialysis vintage was defined as dialysis duration at study entry. For continuous variables, hazard ratios are presented per one-unit increase. CRP, C-reactive protein; CrI, credible interval; HR, hazard ratio.
