# Supplementary material for: Longitudinal trajectories of nutrition-related biomarkers and mortality risk in maintenance hemodialysis patients: a joint modeling analysis
Source: Front Nutr. 2026 Jun 24;13:1769563. doi: 10.3389/fnut.2026.1769563 (PMC13341511; doi:10.3389/fnut.2026.1769563)
Supplement: Supplementary file 3 [file Data_Sheet_3.PDF]

Supplementary Table S3. Dynamic prediction performance of the adjusted joint model using a 12-month prediction horizon

Landmark time, months	Prediction horizon, months	Time-dependent AUC	Brier score
6	12	0.768	0.078
9	12	0.773	0.072
12	12	0.78	0.074
15	12	0.807	0.067
18	12	0.756	0.071
21	12	0.759	0.073
24	12	0.782	0.07
27	12	0.762	0.086
30	12	0.783	0.072
33	12	0.79	0.097
36	12	0.79	0.101

Time-dependent AUC and Brier score were calculated from the adjusted joint model incorporating longitudinal C-reactive protein and serum iron. A fixed 12-month prediction horizon was used across all landmark times. AUC, area under the curve.
